# Molecular Test to Assign Individuals within the *Cacopsylla* *pruni* Complex

**DOI:** 10.1371/journal.pone.0072454

**Published:** 2013-08-19

**Authors:** Jean Peccoud, Gérard Labonne, Nicolas Sauvion

**Affiliations:** INRA, UMR 0325 Biologie et Génétique des Interactions Plantes-Parasites, Campus International de Baillarguet, Montpellier, France; Institute of Biochemistry and Biology, Germany

## Abstract

Crop protection requires the accurate identification of disease vectors, a task that can be made difficult when these vectors encompass cryptic species. Here we developed a rapid molecular diagnostic test to identify individuals of *Cacopsylla* *pruni* (Scopoli, 1763) (Hemiptera: Psyllidae), the main vector of the European stone fruit yellows phytoplasma. This psyllid encompasses two highly divergent genetic groups that are morphologically similar and that are characterized by genotyping several microsatellite markers, a costly and time-consuming protocol. With the aim of developing species-specific PCR primers, we sequenced the Internal Transcribed Spacer 2 (ITS2) on a collection of *C* *. pruni* samples from France and other European countries. ITS2 sequences showed that the two genetic groups represent two highly divergent clades. This enabled us to develop specific primers for the assignment of individuals to either genetic group in a single PCR, based on ITS2 amplicon size. All previously assigned individuals yielded bands of expected sizes, and the PCR proved efficient on a larger sample of 799 individuals. Because none appeared heterozygous at the ITS2 locus (i.e., none produced two bands), we inferred that the genetic groups of *C* *. pruni* , whose distribution is partly sympatric, constitute biological species that have not exchanged genes for an extended period of time. Other psyllid species (*Cacopsylla*, *Psylla*, Triozidae and Aphalaridae) failed to yield any amplicon. These primers are therefore unlikely to produce false positives and allow rapid assignment of *C* *. pruni* individuals to either cryptic species.

## Introduction

Psyllids, or jumping plant lice, are small (1-5 mm) phloem-sucking insects with a worldwide distribution and which breed almost exclusively on perennial dicotyledonous hosts [1–3]. Psyllids typically present very narrow host ranges corresponding to low-level plant taxa, either a specific plant family, genus or species (not considering “shelter plants” on which many psyllid species overwinter) [4]. There are about 3,500 described species of psyllids but twice that many may actually exist [5]. Psyllids belong to the superfamily Psylloidea, a sister clade of all other Sternorrhyncha [6,7].

The taxonomy and phylogeny of psyllids are primarily based on morphological characters (e.g., [8]). Extant jumping plant lice constitute morphologically well-defined groups [9], although, in general, only specialized taxonomists are able to interpret certain subtle characters that distinguish taxa. This is particularly true for the 239 known and validated species of *Cacopsylla* [10], due to the lack of an identification key for the genus [11]. This difficulty becomes problematic for the pests or the vectors of plant pathogens, including *Cacopsylla* species that feed on fruit trees and that transmit bacteria known as phytoplasma. Hodkinson [12] highlighted major problems on the taxonomic status of the various species of west Palaearctic *Pyrus*-feeding psyllids. In their revision, Burckhardt and Hodkinson [1] estimated that European economic entomologists have generally applied the species names, *Cacopsylla* *pyri* (Linné, 1758) and *Cacopsyllapyricola* (Foerster, 1848) (described as vectors of Pear decline phytoplasma in Europe and North America, respectively), to all members of the complex and have failed to recognize morphological and biological differences between the seven known species feeding on *Pyrus*. Confusion is also possible between vectors of Apple proliferation phytoplasma, *Cacopsyllamelanoneura* (Foerster, 1848) and *Cacopsylla* *affinis* (Löw, 1880), which are often found together on their wild host plants ( *Crataegus* ) or on their overwintering shelter plants (conifers). *C* *. pruni* , the only known vector of the European stone fruit yellows phytoplasma on cultivated *Prunus* [13,14], was proved to be a complex of two genetic groups that are not yet distinguished morphologically [15]. The difficulty to identify economically important psyllids with traditional taxonomic characters demonstrates the need for a new approach.

DNA-based identification constitutes a reliable, cost-effective and accessible solution to assign individuals to cryptic species. The mitochondrial gene encoding cytochrome *c* oxidase subunit 1 (COI) has been proposed in the ‘DNA barcoding’ approach as the best marker for a global bioidentification system in animals [16]. The ribosomal DNA (rDNA) second internal transcribed spacer (ITS2) is another frequently used marker. This fast evolving nuclear region has proved its benefits for phylogenetic analysis within genera or for discrimination of cryptic species within complexes (e.g., [17,18]).

The aim of the present work was to develop a molecular identification method to distinguish the individuals of the genetic groups A and B of the *C* *. pruni* complex. A previous population structure analysis based on 310 specimens from western and southern France and northern Spain showed the existence of two clearly differentiated genetic groups that occur sympatrically in most sites [15]. Because no morphological differences have yet been found to distinguish the two groups, the only way to identify them required the use of several microsatellite markers, a costly and time-consuming method [19]. Consequently, in order to assign a greater number of individuals, in particular, to determine the geographical distributions and host ranges of the two groups, we developed an easy and reliable molecular test based on the ITS2 region. This marker offers some advantages over the bar coding gene COI in designing species-specific primers due to the higher G-G content and the presence of insertion-deletions (indels) between species. Such primers were designed to efficiently assign the individuals to each group with a single polymerase chain reaction (PCR) and no DNA sequencing.

## Materials and Methods

### Psyllid samples used in molecular procedures

**Figure 1:**
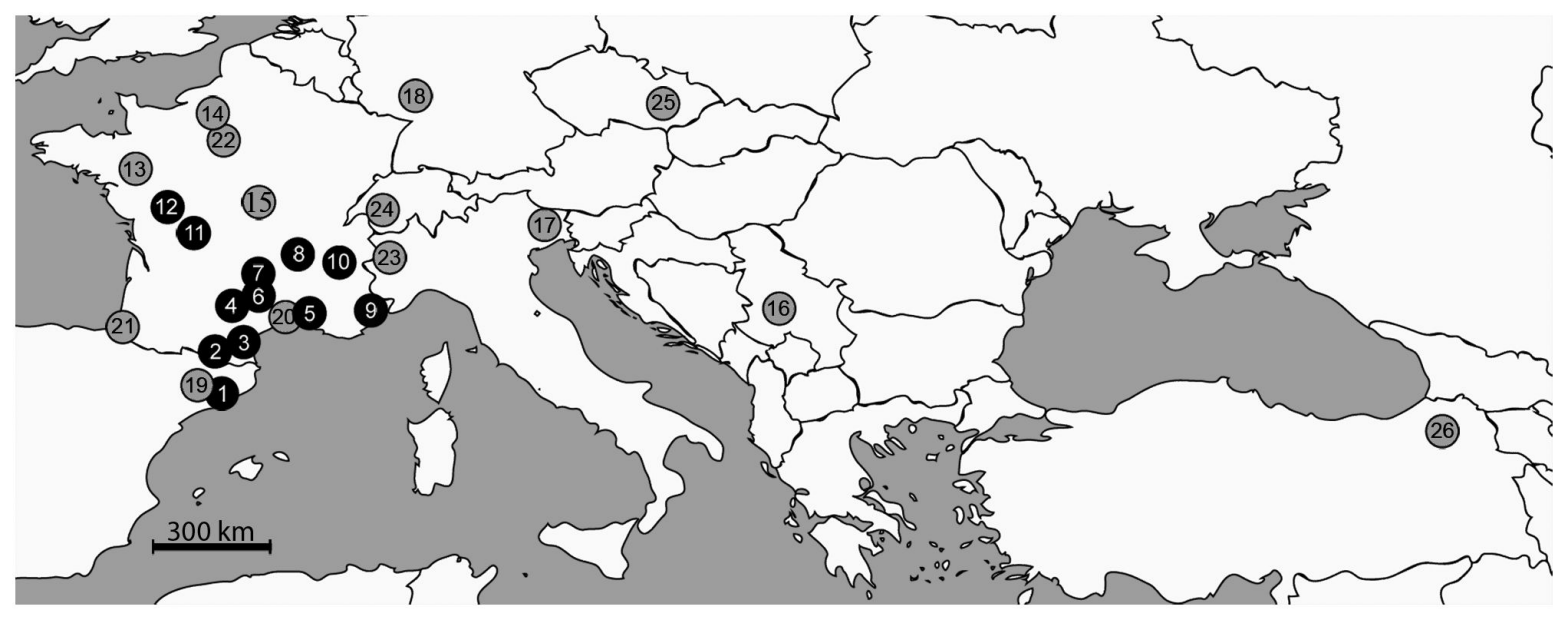
Origin of *C* *. pruni* individuals used in this work. The 12 samples previously genotyped and assigned to groups A or B in Sauvion et al. [15] are shown in black. The 14 samples added to cover the known distribution area of *C* *. pruni* are shown in gray. See Table 1 for details on each location.

Twelve *C* *. pruni* samples (i.e., insects from the same locality) used in this study, mostly from France, are described in Sauvion et al. (2007) (Table 1, Figure 1). These samples constitute 310 psyllids (males or females) genotyped at nine microsatellite loci and previously assigned to groups referred to as *A* and *B* (Table 1). We aimed to increase our dataset with 14 additional samples in order to cover the known distribution area of *C* *. pruni* to the greatest extent possible [14]. The individuals of seven of these 14 samples were genotyped: three samples from France and four samples from Serbia, Italy, Germany and Spain (samples 13 to 19, Table 1). All microsatellite genotypes were assigned to a group using the Bayesian admixture procedure described in Sauvion et al. [15]. In all the 19 genotyped samples, two individuals assigned to either group were selected for ITS2 sequencing (detailed below). As outgroups, we sequenced one individual from *Cacopsyllafulguralis* (Kuwayama, 1908) and one individual from *Psylla alni* (Linné, 1758). Eight individuals of the 19 previously described samples plus seven other samples were analyzed at the diagnostic PCR (see below) to test the reliability of this test on individuals of diverse origins. Our aim was to test four individuals from each group.

**Table 1:** Samples of *C* *. pruni* used in this work.

Sample	Country	Location	Latitude	Longitude	µsat		Sequence		ITS set 1			ITS set 2			ITS set 3				
					A	B	A	B	A	B	null	A	B	null	A	B		null	
1	ES	Tordera	41°46'20.93″N	2°45'33.44″E	9	18	0	1	4	4	0	4	3	1B	4	4		0	
2	FR	Prades	42°37'12.59″N	2°26'8.03″E	28	2	2	2	6	2	0	6	2	0	6	2		0	
3	FR	Torreilles	42°44'28.46″N	2°59'6.05″E	16	10	2	2	4	4	0	4	4	0	4	4		0	
4	FR	Montagne-Noire	43°22'4.43″N	2°18'5.58″E	12	18	2	2	4	4	0	4	4	0	4	4		0	
5	FR	Prades-le-Lez	43°42'17.75″N	3°51'0.78"E	23	7	2	2	4	4	0	4	4	0	4	4		0	
6	FR	Larzac	43°56'57.79″N	3°16'47.71″E	34	0	2	0	8	0	0	8	0	0	8	0		0	
7	FR	La Tieule	44°23'13.02″N	3°7'27.64″E	27	2	2	2	6	2	0	6	2	0	6	2		0	
8	FR	Vesseaux	44°39'9.95″N	4°26'39.62″E	29	3	2	2	5	3	0	5	3	0	5	3		0	
9	FR	Coursegoules	43°47'41.69″N	7°2'18.45″E	21	9	2	2	4	4	0	4	4	0	4	4		0	
10	FR	Romette	44°34'55.18″N	6°6'24.37″E	3	2	0	1	4	4	0	4	4	0	4	4		0	
11	FR	Bellac	46°6'24.58″N	1°4'45.66″E	0	7	0	2	0	7	1B	0	8	0	0	8		0	
12	FR	Montgamé	46°44'1.84″N	0°30'36.72"E	2	28	0	2	2	6	0	2	6	0	2	6		0	
13	FR	Angers	47°29'13.43″N	0°37'9.71"W	12	0	2	0	7	0	1A	8	0	0	7	0		1A	
14	FR	Versailles	48°50'13.79″N	2°1'19.88″E	3	14	2	2	3	5	0	3	5	0	3	5		0	
15	FR	Montmarault	46°21'3.26″N	2°59'21.87″E	0	28	0	2	0	8	0	0	8	0	0	8		0	
16	RS	Čačak	43°53'9.39″N	20°20'45.17″E	0	23	0	2	0	8	0	0	8	0	0	8		0	
17	IT	Udine	46°22'24.01″N	13°8'7.49″E	0	25	0	2	0	8	0	0	8	0	0	8		0	
18	DE	Neustadt	49°24'8.03″N	8°13'6.86″E	0	24	-	-	0	8	0	0	8	0	0	8		0	
19	ES	Moia	41°49'29.78″N	2°11'38.40″E	50	0	2	0	8	0	0	8	0	0	8	0		0	
20	FR	Grabels	43°39'36.73″N	3°49'11.44″E	-	-	-	-	5	3	0	5	3	0	5	3		0	
21	FR	St-Jean PdP	43°14'30.97″N	1°18'6.76″W	-	-	-	-	4	4	0	4	4	0	4	4		0	
22	FR	Farcheville	48°24'37.33″N	2°17'20.11″E	-	-	-	-	4	4	0	4	4	0	4	4		0	
23	IT	Torino	45°7'0.14"N	7°23'43.24″E	-	-	-	-	3	3	1A1B	2	3	2A1B	4	4		0	
24	CH	Martigny	46°7'46.42″N	7°3'46.52″E	-	-	-	-	3	3	1A1B	3	4	1A	4	4		0	
25	CZ	Velešovice	49°10'28.54″N	16°51'39.48″E	-	-	-	-	0	8	0	0	8	0	0	8		0	
26	TR	Erzurum	40°37'14.0″N	41°57'02.22″E	-	-	-	-	0	8	0	0	6	2B	0	8		0	

Columns on the right list the number of individuals of each genetic group (A or B) genotyped at microsatellites (µsat), sequenced at the ITS2 locus (sequence) and tested with each of the three primer sets. The “null” column indicates the number of individuals (A or B) showing no amplification with the diagnostic primer sets. Country codes are defined in ISO 3166-1.

Finally, in order to ascertain that the diagnostic PCR amplified only *C* *. pruni* DNA, we performed it on individuals of 18 species of *Cacopsylla*, including all those known to transmit phytoplasma to Rosaceae, one species of *Psylla*, one species of Triozidae, and one species of Aphalaridae (Table 2), according to the recent classification of Psylloidea [10,11]. Four to five individuals per species were first amplified at the ITS2 locus using generic primers (see below), to ascertain DNA quality at this locus. For each species, one individual that yielded good amplification was then tested with the diagnostic PCR.

**Table 2:** Origin of the 24 psyllid species used in this work.

Sample	Species	Family	Collection plant	Country	Location
1	*Cacopsylla* *affinis* (Löw, 1880)	Psyllidae	Picea abies* (L.) Karst.	CZ	Drahany Highlands (Moravia)
2	*Cacopsyllaalaterni* (Foerster, 1848)	Psyllidae	*Rhamnus* *alaternus* L.	FR	Puéchabon (Hérault, France)
3	*Cacopsylla* *bidens* (Šulc, 1907)	Psyllidae	*Pyrus communis* L.	FR	Montpellier (Hérault, France)
4	*Cacopsyllabreviantennata* (Flor, 1861)	Psyllidae	*Abies* sp*.	FR	Séranne (Hérault, France)
5	*Cacopsyllabrunneipennis* (Edwards, 1896)	Psyllidae	Picea abies* (L.) Karst.	CZ	Drahany Highlands (Moravia)
6	*Cacopsyllacrataegi* (Schrank, 1801)	Psyllidae	Picea abies* (L.) Karst.	CZ	Drahany Highlands (Moravia)
7	*Cacopsyllaelegantula* (Zetterstedt, 1840)	Psyllidae	Picea abies* (L.) Karst.	CZ	Drahany Highlands (Moravia)
8	*Cacopsyllahippophaes* (Foerster, 1848)	Psyllidae	*Prunus* *armeniaca* L.	CZ	Velké Bilovice (Moravia)
9	*Cacopsylla* *mali* (Schmidberger, 1836)	Psyllidae	*Malus* *domestica* Borkh.	BE	Saint-Trond (Limburg, Belgium)
10	*Cacopsyllamelanoneura* (Foerster, 1848)	Psyllidae	*Crataegus* *monogyna* Jacq.	BE	Court St-Etienne (Wallonia, Belgium)
11	*Cacopsylla* *notata* (Flor, 1861)	Psyllidae	*Pyrus* *pyraster* (L.) Du Roi	FR	Les Matelles (Hérault, France)
12	*Cacopsylla* *peregrina* (Foerster, 1848)	Psyllidae	*Picea abies* (L.) Karst.	CZ	Ctyri Dvory (Moravia)
13	*Cacopsylla* *picta* (Foerster, 1848)	Psyllidae	*Malus* *domestica* Borkh.	TR	Hotamis (Mersin, Turkey)
14	*Cacopsylla* *pruni* (Scopoli, 1763) A	Psyllidae	*Prunus* *spinosa* L.	FR	Langon (Gironde, France)
15	*Cacopsylla* *pruni* (Scopoli, 1763) B	Psyllidae	*Prunus* *spinosa* L.	FR	Sauveterre-G (Gironde, France).
16	*Cacopsylla* *pulchella* (Löw, 1877)	Psyllidae	*Cercis* *siliquastrum* L.	FR	Le Pouget (Hérault, France)
17	*Cacopsylla* *pulchra* (Zetterstedt, 1840)	Psyllidae	Picea abies* (L.) Karst.	CZ	Drahany Highlands (Moravia)
18	*Cacopsylla* *pyri* (Linné, 1758)	Psyllidae	*Pyrus communis* L.	FR	Angers (Maine-et-Loire, France)
19	*Cacopsyllapyricola* (Foerster, 1848)	Psyllidae	*Pyrus communis* L.	TR	Antakya (Hatay, Turkey)
20	*Cacopsyllapyrisuga* (Foerster, 1848)	Psyllidae	*Pyrus* *spinosa* Forssk.	FR	Valmascle (Hérault, France)
21	*Cacopsyllarhamnicola* (Scott, 1876)	Psyllidae	Picea abies* (L.) Karst.	CZ	Drahany Highlands (Moravia)
22	*Psylla buxi* (Linné, 1758)	Psyllidae	*Buxus* *sempervirens* L.	FR	La Couvertoirade (Aveyron, France)
23	*Lauritrioza* *alacris* (Flor, 1861)	Triozidae	*Laurus* *nobilis* L.	FR	Le Pouget (Hérault, France)
24	*Ctenarytaina* *eucalypti* (Maskell, 1890)	Aphalaridae	*Eucalyptus* *globulus* Labill.	FR	Porto (Corsica)

Plants marked with an asterisk (conifers) are used by psyllids for overwintering and not for reproduction.

All psyllids were stored in absolute ethanol until DNA extraction. DNA of individual psyllids was purified from whole bodies using either the CTAB (cetyltrimethylammonium bromide) method [20] in individual 1.5 ml tubes, or the High-Salt method based on TNES (Tris, NaCl, EDTA, SDS) buffer [21] in semi-deep well plates. DNA was diluted in 60 µL of water.

### Internal Transcribed Spacer 2 (ITS2) DNA sequencing and phylogenetic analysis

The ITS2 spacer and parts of the rRNA 28S and 5.8S genes were amplified with primers CAS5p8sFcm (5’-CGAACATCGACAAGTCGAACGCACA-3’), adapted by E. Jousselin for aphids (com. pers.) from Ji et al. [22], and CAS28sB1d (ID: S50426), specific for Hemiptera (5’- TTGTTTTCCTCCGCTTATTAATATGCTTAA-3’) [22]. The PCR was performed in a final volume of 25 µL containing: 2.5 µL of QIAGEN Coraload buffer (containing 45 pmol MgCl_2_), 0.05 mM of each dNTP, 3 mM of MgCl_2_ (Qiagen), 0.7 µM of each primer, 0.625 U of *Taq* DNA Polymerase (Qiagen) and 2 µL of DNA extract. The PCR cycles were as follows: initial denaturation at 94°C for 3 min; followed by 40 cycles at 92°C for 30 s, 48°C for 1 min and 72°C for 1 min, with a final 10-min extension period at 72°C. Purification of PCR products and Sanger sequencing with both primers were performed by the GENOSCOPE (Évry, France).

Sequences were analyzed with Geneious Pro 5.4 [23]. Chromatograms from DNA sequences were assembled for each individual and then aligned with the Muscle algorithm and visually inspected. A neighbor-joining tree [24] was constructed using the PAUP* 4.0b10 [25] plugin in Geneious. We used the unequal-frequency Kimura 3-parameter + gamma model of nucleotide evolution [26], as suggested by MODELTEST [27]. Node support was calculated from 1,000 bootstrap replicates.

### Primers for diagnostic PCR

**Figure 2:**
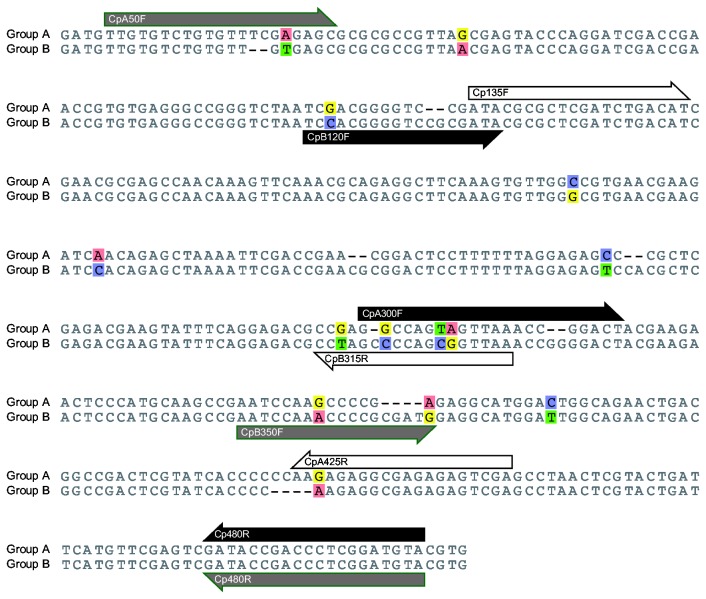
Positions of the primer binding sites in the internal transcribed spacer 2 (ITS2) of *C* *. pruni* groups A and B. Primers are shown in black for set 1, gray for set 2 and white for set 3.

The reciprocal monophyly of the two *C* *. pruni* groups (see results) permitted the development of specific primers. We used the Primer3 web interface (http://bioinfo.ut.ee/primer3/ Accessed 2013 July 19) to design three sets of primers, each comprising a “universal” primer binding the sequence of both groups, and two specific primers, each binding the ITS2 of one group only (Figure 2). This specificity was ensured by the presence of indels that differentiated groups at the primer binding sites. These binding sites were chosen at distant positions so that a triplet of primers made it possible to identify groups by sizing the amplicon produced in a single PCR. In the following, the name of a group-specific primer comprises the letter A or B to specify the genetic group that it targets. For the first set, the universal primer was Cp480R (5’-TACATCCGAGGGTCGGTATC-3’), and the specific primers were CpA300F (5’- GGCCAGTAGTTAAACCGGACT-3’) and CpB120F (5’-TCCACGGGGTCCGCGATA-3’). For the second set, the same universal primer Cp480R was used with the specific primers CpA50F (3’-TTGTGTCTGTGTTTCGAGAGC-5’) and CpB350F (3’-AATCCAAACCCCGCGATG-5’). For the third set, the universal primer was Cp135F (3’- ATACGCGCTCGATCTGACAT-5’), and the two specific primers were CpA425R (3’- TCGACTCTCTCGCCTCTCTT-5’) and CpB315R (3’-TTAACCGCTGGGGCTAGG-5)’.

PCR was performed in a final volume of 12 µL containing: 1.1 µL of QIAGEN Coraload PCR buffer (containing 45 pmol MgCl_2_), 0.05 mM of each dNTP, 2.5 mM of MgCl_2_ (Qiagen), 0.2 µM of each primer (10 µM), 0.625 U of *Taq* DNA Polymerase (Qiagen) and 2 µL of DNA extract. Due to the high yield of this PCR, DNA template was diluted 30 times before being added to the mix. The PCR cycles were as follows: initial denaturation at 94°C for 5 min; followed by 30 cycles at 94°C for 30 sec, 65°C for 20 sec and 72°C for 30 sec, with a final 5-min extension period at 72°C. Amplicons were submitted to a 100V, 40 min electrophoresis in 2% agarose gels stained with ethidium bromide for visualization under UV light.

## Results

### Phylogeny of C. pruni at the ITS locus

**Figure 3:**
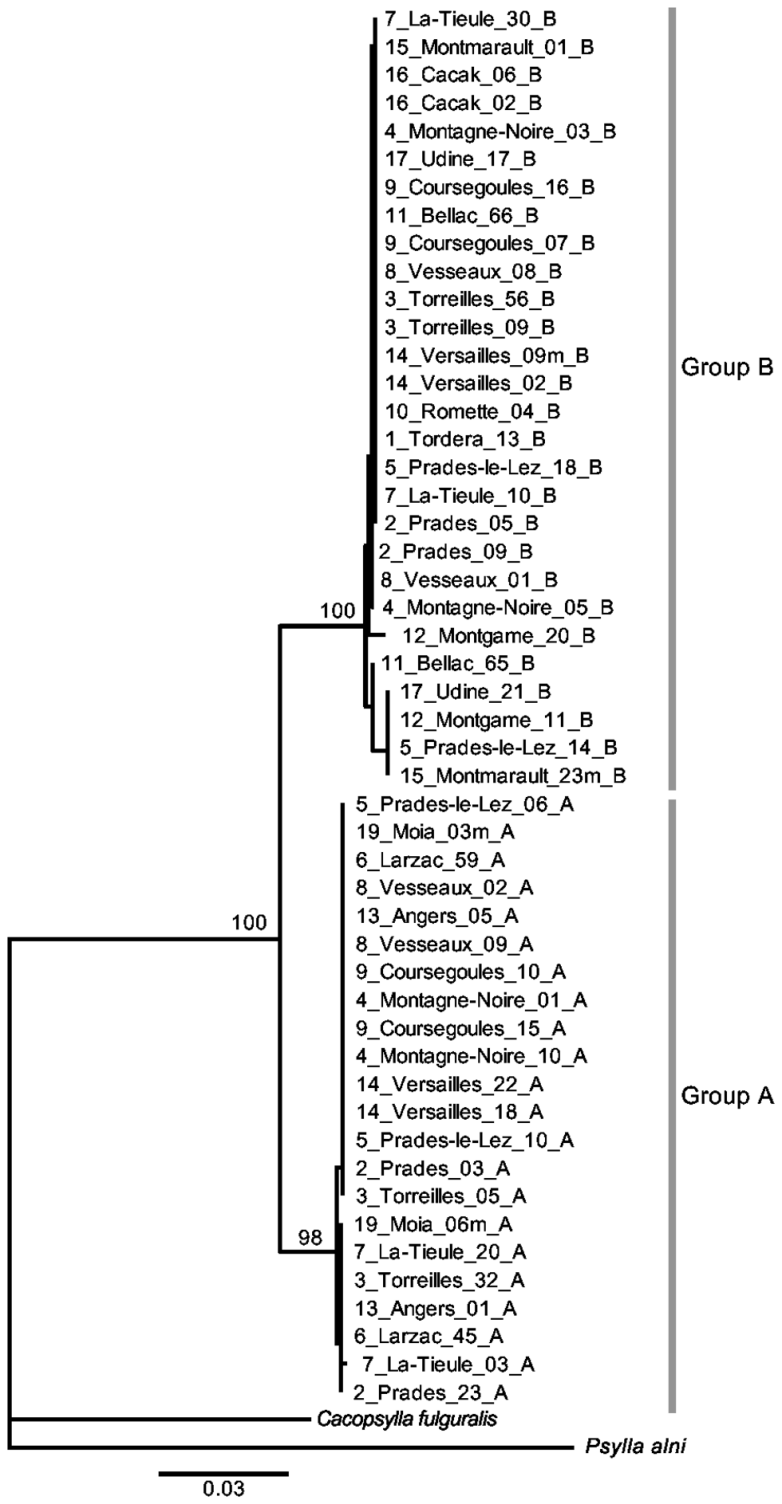
Neighbor-joining phylogeny of ITS2 sequences from individuals of *C* *. pruni* . Tip labels indicate the sample number of each insect (as defined in Table 1), followed by its sampling locality, individual number, sex (m for males) and assignment to genetic group corresponding to microsatellite genotype cluster A or B, according to Sauvion et al. [15]. Bootstrap support is indicated next to nodes when it exceeds 95%.

Figure 3 shows the neighbor-joining tree of ITS sequences from *C* *. pruni* individuals with different origins (two to four individuals tested in 18 samples, Table 1), and from *C* *. fulgaris* and *P* *. alni* (the latter used as the outgroup). The sequencing was unsuccessful for only eight individuals out of the 58 tested, three from Tordera (sample 1, two individuals from group A and one from group B), three from Romette (sample 10, two individuals from group A and one from group B), and two individuals from group A from Montgamé (sample 12). Two highly divergent clades strictly correspond to previously characterized microsatellite genotype clusters, so that each cluster is monophyletic at that locus (Figure 3). Sequence divergence within a clade is low in comparison to divergence between clades. Sequences are available under Genbank accession numbers KF305120 to KF305171.

### Diagnostic PCR

**Figure 4:**
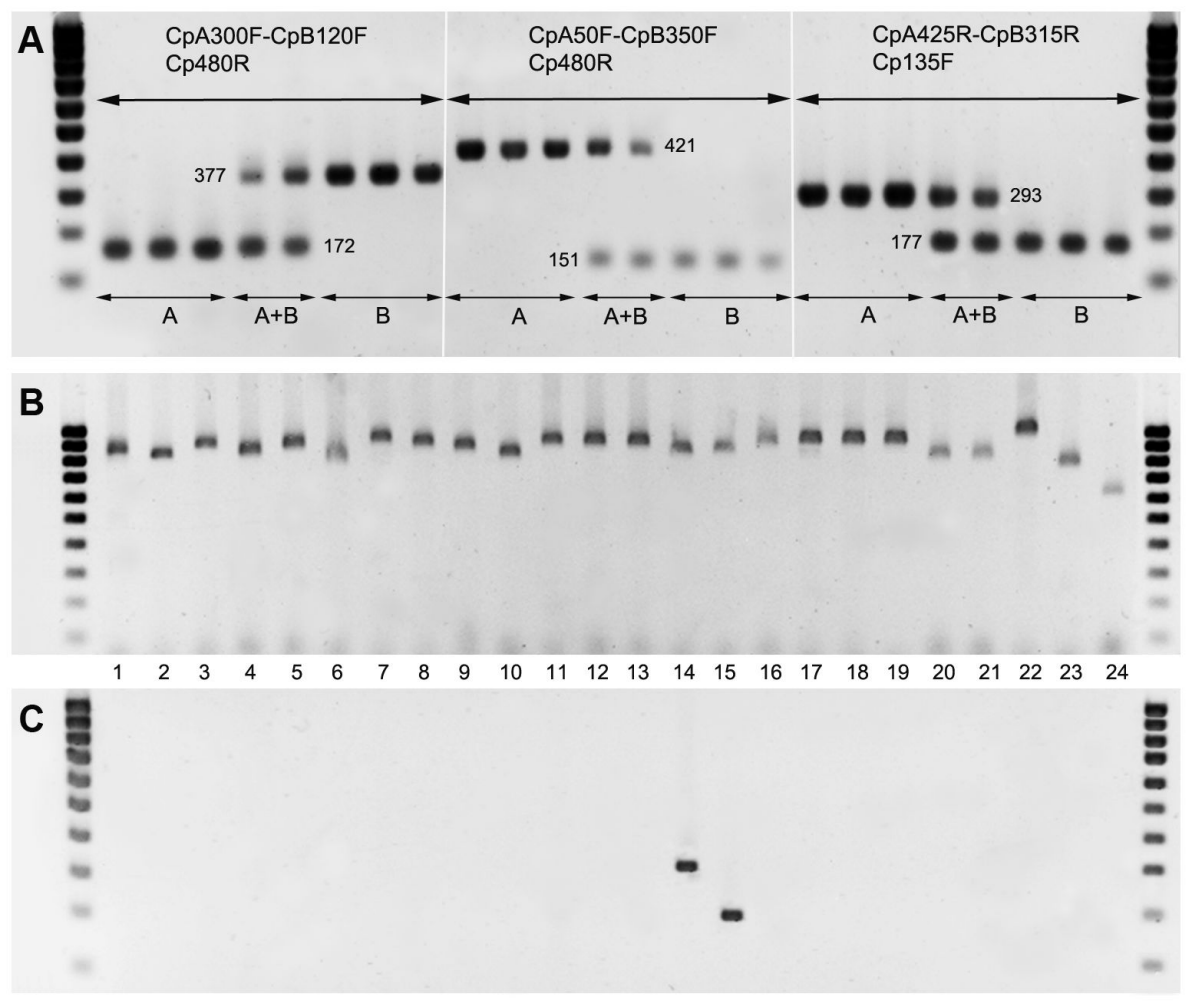
Amplicons from PCRs using the ITS2 primer sets designed for the characterization of *C* *. pruni* individuals. Panel A: typical bands obtained from single individuals belonging to groups A or B, and of pools of two individuals of each group (A+B) with each set of primers. The rightmost and leftmost lanes used a 100 bp DNA ladder. Numbers indicate the expected sizes of the amplicons. Panels B and C: PCRs using generic ITS2 primers FCM/B1D and the primer set 3 (Cp135F / CpB315R / CpA425R), respectively, on single individuals from the 24 psyllid species described in Table 2.

Figure 4A shows typical bands obtained by electrophoresis of PCR products from each *C* *. pruni* group using the three primer sets designed to amplify the ITS2 region. For primer set 1, the expected PCR products are 172 base pairs (bp) for group A and 377 bp for group B. For primer set 2, the expected PCR products are 421 bp (A) and 151 bp (B), and for primer set 3, the expected PCR products are 293 bp (A) and 177 bp (B). The mixtures of group A DNA and group B DNA (holes A+B on Figure 4A) resulted in two bands of the expected sizes.

Eight individuals per sample (males or females assigned to groups A and/or B based on their microsatellite genotype) were tested with the three primers sets. All amplicons obtained were of the expected sizes (Table 1). Seven, eight and two individuals resulted in no band for the primer sets 1, 2 and 3, respectively. For each sample, the remaining individuals (178 from group A and 103 from group B) were tested with at least one primer set. Only 2% produced no fragment in one PCR, but all of the 799 individuals produced expected amplicons in at least one PCR. None produced two bands. The amplification with primer set 2 seems less efficient for the individuals in group B: the fragments appeared to be the faintest with set 2 than with set 1 or 3 for almost all of the individuals that we tested. Amplification of DNA from *C* *. pruni* males and females revealed no difference.

The specificity of the three primer sets for *C* *. pruni* was evaluated by performing PCR assays on individuals of 22 other psyllid species. All were amplified at the ITS2 region with the generic primers (Figure 4B), but none showed amplification with any of the primer sets designed for *C* *. pruni* . Indeed, amplicons were obtained only for the *C* *. pruni* individuals, corresponding to lanes 14 and 15 in Figure 4C, which shows results for primer set 3 (results not shown for primer sets 1 and 2).

## Discussion

The perfect match between microsatellite clusters, ITS2 clades and amplicon sizes demonstrates that ITS2 is a reliable marker to identify groups of *C* *. pruni* that remain undifferentiated morphologically. Because no individuals of the 799 tested produced amplicons of both sizes, there is no evidence for successful hybridization or introgression of ITS2 alleles between the genetic groups often found in sympatry, since such events would lead to heterozygous individuals. This result indicates that the genetic groups of *C* *. pruni* constitute biological species. Furthermore, divergence between ITS2 alleles within each *C* *. pruni* species is much lower than between species (Figure 3). Such deep reciprocal monophyly suggests that the *C* *. pruni* species did not exchange ITS2 alleles for a relatively long time [28]. Absolute estimates on the timing since complete isolation would require a molecular clock, which is not available in psyllids.

The failure to amplify the ITS2 locus in other species with our diagnostic primers probably results from mutations in primer binding sites, which were chosen for their polymorphism within *C* *. pruni* . In these conditions, it is extremely unlikely for an individual that produces one band of a certain size to be erroneously assigned. The fact that some *C* *. pruni* individuals failed to amplify with one given set of primers may result from technical errors or null ITS2 alleles. The use of several sets of primers will ensure successful identification of such individuals.

The diagnostic PCR developed here is a fast, cost-effective and reliable tool to assign individuals of *C* *. pruni* to genetic groups that appear to constitute divergent species. This tool will greatly facilitate studies that investigate the distribution areas of these species, their host plants and their ability to vector phytoplasma pathogens.
